# Influence of Age and Other Factors on Cytokine Expression Profiles in Healthy Children—A Systematic Review

**DOI:** 10.3389/fped.2017.00255

**Published:** 2017-12-14

**Authors:** Marie-Luise Decker, Martin P. Grobusch, Nicole Ritz

**Affiliations:** ^1^Pediatric Infectious Diseases and Pediatric Pharmacology, University of Basel Children’s Hospital, Basel, Switzerland; ^2^Center of Tropical Medicine and Travel Medicine, Department of Infectious Diseases, Division of Internal Medicine, Academic Medical Center, University of Amsterdam, Amsterdam, Netherlands; ^3^Institute of Tropical Medicine, University of Tübingen, Tübingen, Germany; ^4^Department of Pediatrics, The University of Melbourne, Parkville, Australia

**Keywords:** cytokine expression, gender, children, infant, adolescent, sex, age influence

## Abstract

Cytokines have attracted much attention as diagnostic biomarkers for infectious and inflammatory diseases in recent years. However, understanding of maturation and normal age-associated values is limited. This review summarizes evidence on the influence of age and other factors on expression profiles of soluble and intracellular cytokines in healthy children. IFN-γ, IL-6, IL-10, and TNF-α are the most frequently investigated cytokines, of which an age-associated increase was shown consistently for IFN-γ and TNF-α. An age-associated decrease of IL-13 was seen in resource-limited settings. For other cytokines, including IL-1RA, IL-2, and IL-10, uni- or bimodal curves have been described, and results were influenced by study setting. To conclude, despite limited current understanding of the development of cytokine expression, age clearly influences expression profiles in healthy children. Dynamics of cytokine expression in childhood need to be considered when these are measured in diagnostic assays or as biomarkers. In addition, cytokine-targeting agents may require adjustment for normal values when used in children.

## Background

Cytokines play an important role in cell signaling for normal development and in a wide range of diseases (1). Their role as diagnostic and prognostic biomarkers includes infectious, autoimmune, allergic, and hematological diseases. In addition, insight into cytokine profiles in the first few years of life may also inform on optimal immunization strategies. In recent years, measurement of cytokines in pediatric patients has attracted much attention. For diagnostic purposes, cytokines may be measured in unstimulated serum, such as interleukin (IL)-6 in neonatal sepsis or after specific antigen-stimulation such as the interferon-γ release assay (IGRAs) for the diagnosis of tuberculosis. Several studies suggest that the expression of certain cytokines is influenced by age; however, larger datasets on cytokine profiles for healthy neonates, infants and children are lacking (2–6). The aim of this review was to assess the literature on cytokine concentrations in healthy children and to summarise current knowledge on normal cytokine expression and factors that influence normal values in the pediatric age group.

## Search Strategy and Selection Criteria

References for this review were identified through searches of Medline, EMBASE, and Web of Science for articles published between 1974 [when the term “cytokine” was first used in medical literature (7)] and Feb 2017, by use of the medical subject headings “cytokine” and “child” and the search terms “healthy” and “serum.” References of identified articles were searched for additional publications. Only articles with sufficiently detailed data on cytokine analysis and separate analysis of cytokine data in healthy children were included. Studies describing results in cord blood samples only and/or one age group only were excluded. Articles published in English were included.

## Results

### Search Results

**Figure 1:**
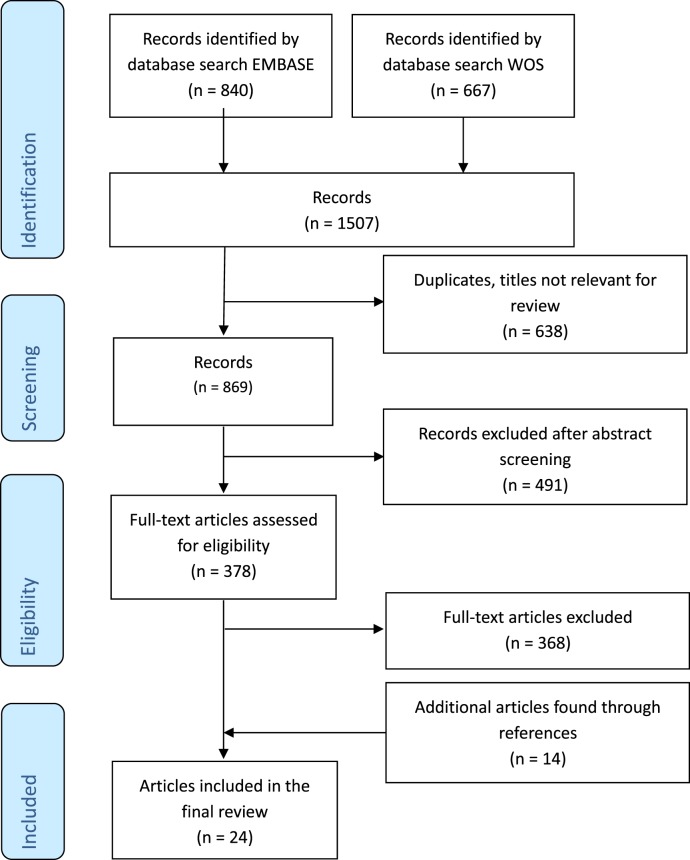
Flow diagram of selected articles included in the review.

The results of the search and selection process are summarized in Figure 1. A total of 1,507 articles were screened, and 24 studies were included in the final analysis. Excluded studies investigated individuals without control groups and/or without sufficient data on healthy control groups. The 24 studies comprised results from 174 cord blood samples, 3,363 samples from children up to 18 years of age and 226 samples from adults (Table 1). Studies originated from 16 different countries, with more than half of the studies originating from five countries (Germany, Australia, Belgium, Italy, and Japan). Seven (29%) studies included participants in resource-limited settings in Brazil, Ecuador, Guinea-Bissau, Indonesia, Papua New Guinea, South Africa, and The Gambia. Four (17%) studies used intracellular cytokine assays (ICA), using flow cytometry only, 18 (75%) studies used non-flowcytometric detection methods [soluble cytokine assays (SCA)] for soluble cytokines in serum or supernatants, and two (8%) studies analyzed both intracellular and soluble cytokines.

**Table 1:** Studies included in final analysis with baseline characteristics, cytokines analyzed and method chosen for analysis.

Reference	Year	Country	*n* Cord blood	*n* Child	*n* Adult	Child age range (years)	% Female (child)	GM-CSF	IFN-γ	IL-1β	IL-1ra	IL-2	IL-4	IL-5	IL-6	IL-7	IL-8	IL-10	IL-12	IL-13	IL-23	IP-10	MCP-1	MIP-1 α	MIP-1β	TNF-α	Other	SCA	ICA
Sack et al. (13)	1997	DE	–	275	–	3–17	–		●		●				●		●									●	^a^	●	
Chipeta et al. (8)	1998	JP	19	16	12	0–14	–		●			●	●																●
Smart and Kemp (19)	2001	AU	–	50	–	0–16	–		●				●	●						●								●	
Upham et al. (20)	2002	AU	30	84	12	0–12	–											●	●									●	
Gasparoni et al. (9)	2003	IT	44	10	10	0–10	–		●			●	●					●											●
Berdat et al. (3)	2003	CH	–	79	–	0.2–4.5	–								●			●								●		●	
Timmons et al. (14)	2005	CA	–	58	–	12–14	43								●		●									●		●	
Härtel et al. (10)	2005	DE	18	54	19	0–8	–		●			●	●	●				●	●							●		●	●
Hoffmann et al. (6)	2005	DE	–	46	33	0–17	–		●			●	●					●								●			●
Okamoto et al. (15)	2005	JP	–	55	44	1–14	35																				TGF-β1	●	
Vaisman et al. (21)	2006	IL	–	21	–	8–12	36			●	●				●			●								●		●	
Yerkovich et al. (11)	2007	AU	10	27	10	0–13	–		●						●			●	●		●					●	^b^	●	●
Eriksson et al. (22)	2007	GW	–	50	11	0.4–0.5	44		●					●				●								●		●	
Wiegering et al. (2)	2009	DE	25	92	–	0–18	42		●			●	●													●			●
Figueiredo et al. (23)	2009	BR	–	1356	–	4–11	46		●					●				●		●								●	
Raes et al. (16)	2010	BE	–	202	–	0.2–0.5	43		●			●	●	●				●								●		●	
Nguyen et al. (24)	2010	BE	13	30	30	0–1	60		●	●					●		●	●	●			●					MIG	●	
Teran et al. (25)	2011	EC	–	240	–	0.5–5	52		●					●	●		●	●		●						●		●	
Burl et al. (26)	2011	GM	15	105	–	0–1	–		●	●					●			●								●		●	
Lisciandro et al. (27)	2012	PG	–	67	–	0.1–1.5	–		●	●					●			●								●		●	
Reikie et al. (12)	2012	SA	–	28	10	0–1	–		●						●		●	●	●		●	●	●	●	●	●	IFN-α	●	
Kleiner et al. (17)	2013	IT	–	37	35	1–17	59	●	●	●	●	●	●	●	●	●	●	●	●	●	●	●	●	●	●	●	^c^	●	
Djuardi et al. (28)	2016	ID	–	119	119	0.2–2	–		●					●				●		●						●		●	
Dorn et al. (18)	2016	US	–	262	–	11–17	100	●	●	●		●	●	●	●	●	●	●	●	●		●				●		●	
Total			174	3244	226			2	19	6	3	8	9	8	12	2	8	18	7	6	3	4	2	2	2	18		20	6

*SCA, soluble cytokine assays (i.e., in serum or supernatants); ICA, intracellular cytokine assay measured using flow cytometry*.

*^a^Neopterin, sCD14, sE-selectin, sICAM-1, sIL-2r, sTNF-RII*.

*^b^IL-18, myxovirus resistance protein A*.

*^c^IL-9, IL-13, IL-15, IL-17, Eotaxin, Basic FGF, G-CSF, PDGF-BB, RANTES, TNF-β, VEGF, IL-1α, IL-2Rα, IL-3, IL-16, IL-18, CTACK, GRO-α, HGF, IFN-α2, LIF, MCP-3, M-CSF, MIF, MIG, β-NGF, SCF, SCGF-β, SDF-1α*.

### Studies Investigating Intracellular Cytokines

A study including 19 term newborns, 16 children (mean age 11.6 years, range 9–14 years), and 12 adults (mean age of 36.8 years, range 30–53 years) in Italy stimulated cord blood mononuclear cells (CBMCs) or peripheral blood mononuclear cells (PBMCs) with phorbol-12-myristate-13-acetate (PMA)/ionomycin for 12 h (8). The study did not specify results in unstimulated samples but stated that the proportion of IFN-γ-, IL-2-, and IL-4-producing T cells was “routinely less than 1.0%.” In stimulated samples neonates and children had significantly lower proportions of IFN-γ-producing CD4 and CD8 T cells compared to adults (Tables 2 and 3). For IL-2 producing CD4 T cells proportions were comparable in children and adults, but higher in neonates, and IL-2-producing CD8 T cells were highest in neonates and lower in children and adults (Table 3).

**Table 2:** Proportions of cytokine-producing cells in stimulated CD4 T cells from healthy neonates and children (flow-cytometry data).

			Age (years)																		
Reference	Cord blood preterm	Cord blood term	0	1	2	3	4	5	6	7	8	9	10	11	12	13	14	15	16	17	Adults
**IFN-γ-producing CD4 T cells (%)**																					
Chipeta et al. (8)		0.2										3.8									7.3
Gasparoni et al. (9)	1.0–1.6	0.8				4.7															10.9
Hoffmann et al. (6)			3.7		13.9				11.6							18.7					21.6–23.2
Wiegering et al. (2)		4.0	8.0		11.0					13						17					
**IL-2-producing CD4 T cells (%)**																					
Chipeta et al. (8)		56.4										41.2									42.4
Gasparoni et al. (9)	61.2–54.0	51.3				21.3															31.5
Hoffmann et al. (6)			4.9		9.7				8.6							13.2					12.4–62.8
Wiegering et al. (2)		71	14		29					26						37					
**IL-4-producing CD4 T cells (%)**																					
Gasparoni et al. (9)	0.4–0.6	0.4				1.3															1.6–4.2
Hoffmann et al. (6)			0.5		1.4				1.5							1.7					21.6–23.2
Wiegering et al. (2)		0.6	1.0		1.0					1.0						1.0					
**IL-10-producing CD4 T cells (%)**																					
Gasparoni et al. (9)	0.9	0.4				–															10.9
Hoffmann et al. (6)			0.2		0.5				1.1							1.1					1.3–1.7
**TNF-α-producing CD4 T cells (%)**																					
Hoffmann et al. (6)			3.7		13.9				11.6							18.7					21.6–23.2
Wiegering et al. (2)		4.0	17.0		22.0					24.0						25.0					

**Table 3:** Proportions of cytokine-producing cells in stimulated CD8 T cells from healthy neonates and children (flow cytometry data).

			Age (years)																		
Reference	Cord blood preterm	Cord blood term	0	1	2	3	4	5	6	7	8	9	10	11	12	13	14	15	16	17	Adults
**IFN-γ-producing CD8 T cells (%)**																					
Chipeta et al. (8)		2.8										16.6									36.1
Gasparoni et al. (9)	3.6–4.6	4.6				20.0															27.7
Hoffmann et al. (6)			10.6		30.3				28.1							34.6					38.9–55.8
Wiegering et al. (2)		9.0	21.0		13.0					15.0						22.0					
**IL-2-producing CD8 T cells (%)**																					
Chipeta et al. (8)		18.7										14.3									8.5
Gasparoni et al. (9)	18.9–37.0	23.2				12.1															13.9
Hoffmann et al. (6)			1.2		0.3				1.3							0.4					1.8–4.8
Wiegering et al. (2)		50.0	18.0		18.0					18.0						25.0					
**IL-4-producing CD8 T cells (%)**																					
Gasparoni et al. (9)	1.4	1.7				0.7															0.5
Hoffmann et al. (6)			0.1		0.1				0.7							0.1					0.2–1.4
Wiegering et al. (2)		1.0	1.0		2.0					1.0						1.0					
**IL-10-producing CD4 T cells (%)**																					
Gasparoni et al. (9)	1.1–2.0	2.2				–															–
Hoffmann et al. (6)			0.04		0.04				0.04							0.04					0.1–0.2
**TNF-α-producing CD8 T cells (%)**																					
Hoffmann et al. (6)			4.7		12.9				10.3							17.0					18.1–56.5
Wiegering et al. (2)		7.0	21.0		20.0					26.0						23.0					

Another similar study from Italy included 12 very preterm, 12 preterm and 20 term newborns, 10 older children (aged 3–10 years) and 10 adults (age not specified) and stimulated CBMCs or PBMCs with PMA/ionomycin for 4 h (9). Data from unstimulated samples were not reported. In stimulated samples, all newborns (regardless of gestational age) had significantly lower proportions of IFN-γ-producing CD4 T cells compared to older children and adults (Table 2). Proportions of IL-4-producing CD4 T cells were also lower in newborns compared to older children and adults. Contrary to this, proportions of IL-2-producing CD4 T cells were inversely related to age with highest proportions in preterm and newborns and lowest proportion in children and again increasing proportions in adults. Similar findings were seen in IL-2 and IFN-γ-producing CD8 T cells (Table 3). Within neonates, gestational-age-associated decreases in IFN-γ- and IL-10-producing CD4 T cells and IL-2-producing CD8 T cells as well as an increase in IL-10-producing CD8 T cells were found.

A study including 18 newborns, 54 infants and children (aged 0–8 years), and 19 adults (mean age not specified) in Germany stimulated fresh cord blood or whole blood with PMA/ionomycin or LPS for 5–24 h depending on the cytokine of interest (10). Data from unstimulated samples were not reported. Mean proportions of cytokine-expressing T cells in supernatants were not specified but an age-associated increase for IFN-γ- and TNF-α-producing T cells was seen in the first year of life. IL-12-producing monocytes were also increasing with age.

Another study from Germany including 46 children and 33 adults (mean age not specified) stimulated whole blood with PMA/ionomycin for 6 h (6). Data from unstimulated samples were not reported. In stimulated samples, an age-associated increase of IFN-γ-producing CD4 and CD8 T cells was shown (Tables 2 and 3). Further age-associated increases in proportions of IL-2-, IL-4-, and TNF-α-producing CD4 T cells and for IFN-γ- and TNF-α-producing CD8 T cells were noted (Tables 2 and 3).

A further study from Germany including 117 individuals (neonates, infants, children, and adolescents up to 18 years of age) stimulated fresh cord blood or whole blood with PMA/ionomycin for 24 h (2). An age-associated increase for IFN-γ- and TNF-α-producing CD4 and CD8 T cells was found (Tables 2 and 3). In addition, the study showed a decrease in IL-2-producing CD4 and CD8 T cells with age (Tables 2 and 3). The study was the first to further investigate sex influence on cytokine expression showing higher IL-2 expression in males compared to females in the first 6 years of life. Females, however, had more IL-2-producing cells in the 6- to 12-year group and more INF-γ-producing cells in the 12- to 18-year group compared to their male peers.

Two studies focused on intracellular cytokines in antigen presenting cells. The first one investigated the capacity of monocytes to secrete cytokines in response to IFN-γ priming and LPS stimulation at birth (cord blood) and in adults (median age 31, range 23–57 years) in Australia (11). Mean proportions of IL-6- and TNF-α-producing monocytes were higher in cord blood samples than in samples from adults with 5.1% and 2.6%, and 4.5% and 2.2%, respectively. The second study analyzed intracellular cytokines in monocytes and dendritic cells in 28 infants and 10 adults (aged 24–47 years) in South Africa (12). The infants had several follow-up appointments at 0.5, 1.5, 3, 6, and 12 months of age. Whole blood samples were stimulated with TLR2/1, 2, 3, 4, 7/8, and 9 agonists and with NOD 1/2 receptor agonist for 6 h or were left unstimulated. Intracellular cytokine expression of monocytes and dendritic cells showed an age-associated decrease in the first year of life with the exception of IL-6 and TNF-α after stimulation with LPS (TLR 4 agonist).

Age correlation of studies investigating intracellular cytokines are summarized in Table 4.

**Table 4:** Age correlation of cytokine expression in flowcytometry studies.

	Chipeta et al. (8)	Gasparoni et al. (9)	Härtel et al. (10)	Hoffmann et al. (6)	Yerkovic et al. (11)	Wiegering et al. (2)	Reikie et al. (12)
Sample type	CBMC and PBMC	Frozen CBMC and PBMC	Whole blood from cord and venous samples	Whole blood	Frozen CBMC and PBMC	Whole blood from cord and venous samples	Whole blood

Stimulant	PMA/ionomycin	PMA/ionomycin	LPS PMA/ionomycin	PMA/ionomycin	LPS	PMA/ionomycin	TLR-agonist 1–4, 7–8, NOD-receptor agonist 1/2

Duration of stimulation (h)	12	4	5 (IFN-γ, IL-12, TNF-α) 10, 18, 24 (IL-12)	6	24	24	6

INF-γ	CD4	CD4	CD3	CD4		CD4	–
	CD8	CD8		CD8		CD8	

IL-2	CD4	CD4	–	CD4		CD4^b^	–
	CD8	CD8		CD8		CD8^b^	

IL-4	ULQ	CD4	–	CD4		CD4	–
		CD8		CD8		CD8	

IL-6					CD 14 (monocytes)		Monocytes
							Dendritic cells

IL-10	–	CD4^a^	–	CD4		–	–
		CD8^a^		CD8			

IL-12	–	–	CD14	–		–	–

TNF-α	–	–	CD3	CD4	CD14 (monocytes)	CD4	Monocytes
				CD8		CD8	Dendritic cells

*Negative age correlations are shown in red, positive age correlations in green, and absence of age correlations in gray. Only parameters investigated in more than one study are listed*.

*–, not analyzed; CBMC, cord blood mononuclear cell; PBMC, peripheral blood mononuclear cell, LPS, lipopolysaccharide, PMA, phorbol myristate acetate, ULQ, under the lower limit of quantification*.

*^a^Only for preterm and term newborns (GA 25–39 weeks)*.

*^b^With exception of cord blood samples with significantly higher IL-2 producing cells*.

### Studies Investigating Soluble Cytokines in Unstimulated Samples Only

The first study investigating cytokine expression in healthy children is a German study including 275 children aged 3–17 years, which analyzed cytokine and other soluble marker concentrations in cryopreserved serum samples (13). For five analytes (IL-1RA, soluble CD14, soluble IL-2 receptor, soluble ICAM-1, and sE-selectin) a significant age-associated decrease was seen. IL-6 had a bimodal age-associated pattern peaking at 3-4 and at 15 years of age and TNF-α; an unimodal pattern peaking at 13–14 years of age; and a subsequent decline to adult concentrations. Concentrations of IFN-γ and IL-8 were below the lower limit of quantification.

Another study including 79 children (aged 0 to 5 years) analyzed cytokine concentration in cryopreserved serum using ELISA (3). Mean cytokine concentrations for IL-6, IL-10, and TNF-α were low, with 1.6–9.2 pg/ml, 3.3–5.5 pg/ml, and 2.2–3.5 pg/ml, respectively. Overall, no age association was found, but a trend towards higher TNF-α and lower IL-6 concentrations in children below 2 years of age compared to older children was seen.

A study from Canada including 58 adolescents aged 12–14 years investigated the effect of exercise on cytokine expression (14). Cryopreserved plasma collected at rest was analyzed using ELISA. Mean pre-exercise concentrations for IL-6, IL-8, and TNF-α were low, with 1.0–1.3 pg/ml, 4.0–6.3 pg/ml, and 0.6–0.9 pg/ml, respectively. Age or sex differences at rest were not analyzed. After 60 min of exercise and a further 60 min of recovery small and in few instances significant increases of concentrations were found in all age groups.

A study investigated transforming growth factor (TGF)-β1 in cryopreserved serum samples using ELISA in 55 children (aged 0–14 years) and 44 adults (aged 21–67 years) in Japan as a potential biomarker for Kawasaki disease (15). Mean TGF-β1 was high in both children and adults (62,000 pg/ml and 40,000 pg/ml, respectively) and showed a significant negative correlation with age.

A study including 202 infants in Belgium investigated cytokine expression in cryopreserved serum samples at 2 and 6 months of age using cytometric bead arrays with particular interest on the effect of supplementation of infant formula with prebiotic oligo-saccharides (16). Average concentrations for the non-intervention groups at 2 months of age were 38.8 pg/ml, 9.6 pg/ml, 13.6 pg/ml, 4.3 pg/ml, 12.3 pg/ml, and 7.0 pg/ml for IFN-γ, IL-2, IL-4, IL-5, IL-10, and TNF-α, respectively. At 6 months of age the concentrations were comparable for most cytokines. The study did not demonstrate any changes of cytokine expression in the intervention group.

A study from Italy including 37 children from 1 to 17 years (7 children below 7 years of age) and 35 adults (aged 21–86 years) examined cytokine concentrations using multiplex bead-based immunoassays in serum samples that were cold stored before analysis (17). Concentrations of 16 cytokines showed an age association, of which six were significantly higher in both pediatric groups compared to adults, namely growth-related oncogene (GRO)-α, IL-18, macrophage migration inhibitory factor (MIF), macrophage inflammatory protein (MIP)-1β, stem cell factor (SCF), and stem cell growth factor (SCGF)-β. In contrast, cytokine concentrations of eotaxin and IL-17 were of significantly lower levels in both pediatric groups compared to adults. For five cytokines (IL-4, IL-6, IFN-γ, PDGF-BB, and TNF-α), unimodal patterns were seen with higher concentrations in children aged 7–17 years compared to adults and young children. For IL-13, a unimodal pattern with lowest concentrations in the group of 7- to 17-year-old children compared to adults and young children was seen. Concentrations of 20 cytokines were not influenced by age, and 11 cytokines were below lower limit of quantification [β-NGF, IFN-α, IL-1α, IL-1β, IL-3, IL-5, IL-12 (p40), IL-15, LIF, MCP-3, and TNF-β]. Sex was not found to influence cytokine expression.

A study including 262 adolescent girls (aged 11–17 years) in the US measured cytokine concentrations using multiplex bead-based immunoassays in cryopreserved plasma (18). A high number of samples had concentrations below LLOQ, which was 52% for IL-4, 37% for IFN-γ, and 23 to 27% for IL-12 (p40), IL-13, and GM-CSF. Using a latent profile analysis, the study described four profiles of individuals labeled as “low overall,” “higher innate,” “higher adaptive,” and “high overall” comprising 45%, 21%, 26%, and 7% of all study participants, respectively. Girls in the “high overall” and the “higher innate” groups were younger than the girls in the other ones.

### Non-Flowcytometric Studies Using Unstimulated Samples and/or Stimulated Samples

A study including 50 children (0–16 years, mean age not specified) in Australia stimulated PBMCs with SEB for 48 h (19). In SEB-stimulated samples an age-associated increase was shown for IFN-γ, IL-4, and IL-5, but not for IL-13. Interestingly, the study also analyzed the results in atopic children with a strikingly similar age association.

Another study including 30 cord blood samples, 29 samples from 5-year-old children, 55 samples from 12-year-old children, and 12 samples from adults in Australia stimulated PBMCs with LPS or heat-killed *Staphylococcus aureus* for 24 h, after priming with IFN-γ for 3 h (20). In LPS-stimulated samples, mean concentrations of IL-12p70 were progressively increasing with age with 64.1 pg/ml in cord blood, 311.9 pg/ml in 5-year olds, 778.7 pg/ml in 12-year olds, and 1,922 pg/ml in adults. Similar results were also seen in heat-killed *S. aureus*-stimulated samples. IL-10 concentrations were significantly higher in cord blood samples compared to children and adults.

One of the two studies analyzing both intracellular cytokines and concentrations in supernatants was a German study discussed above (10). Whole blood was stimulated with PMA/ionomycin, or left unstimulated. In stimulated samples, an age-associated increase of cytokine concentrations was seen for IL-2, IL-4, and TNF- α, but not for IL-5 and IL-10. Results for IFN-γ concentrations in supernatants were unfortunately not specified (Table 4).

A study including 21 children (aged 8–12 years) in Israel stimulated PBMCs with LPS for 24 h (21). Mean concentrations of IL-1RA, IL-6, IL-10, and TNF-α in unstimulated samples from control patients were 20 pg/ml, 1.7 pg/ml, 8 pg/ml, and 142 pg/ml, respectively. After stimulation with LPS mean concentrations of all measured cytokines increased by 10-fold to 100-fold. Age or sex influences were not identified.

Another study including 10 samples each from cord blood, or venous blood taken from children at 2 months, 1 year, 4 years, 13 years of age, and from adults (median age 31, range 23–57 years) in Australia, primed CBMCs and PBMCs with IFN-γ for 3 h before stimulation with LPS for 24 h (11). Concentrations of LPS-stimulated samples of IL-6, IL-10, and TNF-α were positively associated with age. However, cord blood samples were exceptional, as high concentrations were found for all three cytokines, reaching similar levels to those seen at 13-year of age and in adults.

One of the few studies performed in resource-limited setting is a study from Guinea-Bissau including 50 infants (4–5 months) and 11 adults (median age 39 years, range 27–61 years) (22). A venous or capillary blood sample was used in a whole blood assay stimulating with TLR2 and 4-agonist (LPS), purified protein derivate (PPD), or PHA; or left unstimulated for 1–3 days. This is one of the few studies with separate results reported from unstimulated samples, showing that IL-10 and TNF-α in the infant group was higher compared to adults, with concentrations of 28 pg/ml and 82 pg/ml and 5 pg/ml and 10 pg/ml, respectively. After stimulation, concentrations for these two cytokines remained higher in the infants compared to adults for all stimulatory antigens except PHA (Table 5). Infants’ cytokine concentrations of IFN-γ and IL-5 were higher compared to adults when stimulated with PHA but lower when stimulated with PPD. Interestingly, the study also showed differences in cytokine concentrations using venous and capillary blood samples, further highlighting the importance of the pre-analytical setting.

**Table 5:** Summary of results in studies reporting age association of secreted cytokines using stimulated samples.

Reference	Smart and Kemp (19)	Upham et al. (20)	Härtel et al. (10)	Yerkovich et al. (11)	Errikson et al. (22)	Figueiredo et al. (23)	Nguyen et al. (24)	Teran et al. (25)	Burl et al. (26)	Lisciandro (27)	Reikie (12)	Djuardi (28)
Age (years)	0–16	0–12, Adult	0–8, Adult	0–13, Adult	0.4–0.5, Adult	4–11	0–1, Adult	0.5–5	0–1	0.1–1.5	0–1, Adult	0.2–2, Adult

Sample type	PBMC	CBMC, PBMC	WB	CBMC, PBMC	WB	WB	WB	WB	WB	WB	WB	WB

Analysis method	ELISA	ELISA	CBA	TRF	xMAP	ELISA	xMAP	ELISA	xMAP	xMAP	xMAP	xMAP

Stimulant	SEB	LPS, KSA	PMA/iono	LPS	TLR2-agnoist LPS, PPD, PHA, LPS	PWM	LPS, TLR2-agonist, CpG	TLR-agonist 1–9, SEB	TLR-agonist 1–9, PMA/iono	TLR-agonist 2, 3, 4, 7/8, NOD 1, 2 ligand, Alum, LPS	NOD 1/2, TLR-agonist 1–4, 7, 8	PHA

Incubation time (h)	24	24	5, 10, 18, 24	2, 6, 24	24, 72	24, 120	16–18	24, 120 (SEB)	18–24	24	18	24, 144

INF-γ		–	–		PHA		LPS	SEB	TLR 1/2	TLR2, 3	All antigens	
					PPD				TLR 3–9	Alum, NOD 1, 2, TRL4, 7/8		

IL-4		–		–	–	–	–	–	–	–	–	

IL-5		–		–	PHA		–	SEB	–	–	–	
					PPD					–		

IL-6	–	–	–		–	–	LPS, CpG	TLR4, 6	TLR9	TLR2, 3	NOD 1/2, TLR2/1, 7/8	
								TLR1/2, 3, 5, 9	TLR1–8	Alum, NOD 1, 2, TLR4, 7/8	TLR3, 4, 9	

IL-8	–	–	–	–	–	–	LPS	TLR1/2, 4, 5, 6, 9	–	–	–	
								TLR 3				

IL-10	–				LPS, PPD, PHA		LPS	SEB, TLR 1/2, 4, 5, 6	TLR3, 9	TLR3	NOD 1/2, TLR2/1	
									TLR7, 8	Alum, NOD 1, 2, TLR3, 4, 7/8	TLR 2/1, 3, 4, 7/8, 9	

IL-12(p70)	–			–	–	–	LPS	–	–	TLR3	–	–

IL-13		–	–	–	–		–	SEB	–	–	–	

TNF-α	–	–			LPS, TLR2	–	LPS	TLR6, 9	TLR 7/8, 9	All antigens	NOD 1/2, TLR2/1	
					PHA			TLR 1/2, 3, 4, 5	TRL8			

*Negative age correlations are shown in red, positive age correlations in green, absence of age correlation (or not analyzed) in gray. Only parameters investigated in more than one study are listed*.

*CBA, cytometric bead array; KSA, heat-killed S. aureus; PWM, pokeweed mitogen; TLR, toll-like receptor; TRF, time resolved fluorometry; xMAP, multiplex bead-based immunoassay; CBMC, cord blood mononuclear cell; PBMC, peripheral blood mononuclear cell; PMA, phorbol-12-myristate-13-acetate; CPG, CpG-rich oligonucleotides*.

The largest study investigating cytokines in healthy children is a Brazilian study including 1,376 children (aged 4–11 years) (23). Whole blood was stimulated with pokeweed mitogen (PWM) or left unstimulated and measured on days 1 and 5. The study unfortunately does not specify results from stimulated or unstimulated samples separately; however, it is stated that only a proportion of children included had detectable concentrations of cytokines being 11.4%, 5.5%, 8.2%, and 34.5% for IFN-γ, IL-5, IL-10, and IL-13, respectively. A negative age correlation was seen for IL-13 but not for any other cytokine measured. Sex differences were only seen for IL-10, with boys having higher concentrations compared to girls. Interestingly, the study also showed that IL-5 and IL-10 concentrations were higher in households with poor sanitation conditions.

A study including 43 infants, in which cord blood and venous blood samples taken at 3, 6, 9, or 12 months and venous samples from 30 adults (mean age 29) stimulated whole blood with LR2 and TLR4 (LPS)-agonists, and with CpG-rich oligonucleotides (CPGs) for 16–18 h (24). In LPS-stimulated samples, concentrations of IFN-γ, IL-12p70, IP-10, and TNF-α showed an age-associated increase with adult concentrations reached by 3 to 6 months of age except for IFN-γ. Other cytokines showed no age association or unimodal distributions. CPG-stimulated samples showed an age-associated increase for IP-10 and monokine induced by gamma interferon (MIG).

A study including 240 children in age groups of 6–9 months, 2 years, and 5 years in Ecuador stimulated whole blood with SEB (5 days) and TLR-agonists 1–8 (24 h) (25). In SEB-stimulated samples, an age-dependent decrease of IL-10 and an increase in IL-5 was found, with most of this effect being observed between the age groups of 6–9 and 22–26 months. After TLR stimulation, different patterns of age-dependent cytokine decrease were seen particularly for IL-8 and IL-10 (Table 5). None of the cytokines showed age-dependent increases after TLR stimulation.

A study including 120 infants (0–12 months) in the Gambia of which 12–15 samples were from cord blood-stimulated whole blood with PMA/ionomycin or TLR-agonists 1–9 for 24 h (26). The largest age-associated differences in cytokine concentrations were seen between results from cord blood samples and those from older infants. After exclusion of cord blood results, only few age- and stimulant-associated changes were found (Table 5).

Another study from a resource-limited setting originates from Papua New Guinea, includined 67 infants (aged 1–18 months) (27). Whole blood was stimulated with TLR-2, 3, 4, 7/8-agonists, nucleotide-binding oligomerization domain (NOD) 1, 2 ligands and aluminum potassium sulfate (Alum) as in the presence and absence of IFN-γ or LPS for 24 h. A significant age-associated increase was shown for INF-γ, IL-1β, IL-6, and IL-10 after TLR2 and TLR3 stimulation. In addition, a trend towards increasing IL-12 concentrations was observed after TLR3 stimulation.

As mentioned above a study from South Africa included flow cytometry analysis but also investigated cytokine expression in culture supernatants (12). The complex analysis performed demonstrated age- and stimulant-dependent decrease in cytokine concentrations for almost all cytokines and stimulants, which reach adult concentrations by 12 months of age. In addition, some cytokine and unimodular curves with peak concentrations around 6 months of age such as IFN-γ, IP-10, and MIP-1α for certain stimulatory antigens.

Another study from Indonesia included 119 mother-child pairs and investigated blood samples serially at 2, 5, 12, 24, and 48 months of age in the children (28). Whole blood was stimulated with PHA for 24 h and 6 days or left unstimulated. The number of children analyzed at each time point decreased from 111 at 2 months to 86 at 2 years of age. An age-associated increase was seen for concentrations of IFN-γ, IL-5, and IL-10 mainly, provided the increase occurred after the first year of life. IL-13 showed an age-associated decrease.

## Discussion

This review represents the most comprehensive summary of currently available data on cytokine profiles and ontogeny of cytokine expression in healthy infants, children, and adolescents up to date. The results display an enormous heterogeneity in the study design limiting conclusions in many instances, and prohibiting a meta-analysis of data. The variability in the participant selection including age range and origin of the participants, as well as the methods of cytokine detection including the sample type, antigens and duration of stimulation, and cytokines analyzed, are considerable.

A number of trends can be observed. Only few studies investigated intracellular cytokines, most likely because this method is more labor- and cost intensive. As a result, studies published since 2009 focus on analysis of soluble cytokines including an increasing number of cytokines, which has become possible with the availability of multiplex assays that allow detection of high numbers of cytokines in a small volume. Despite this, the most frequently investigated cytokines remain IFN-γ, IL-6, IL-10, and TNF-α. Their inclusion in almost all studies relates to their key function in many infectious and inflammatory pathways as well as their potential of being included into diagnostic assays or as drug targets. More recent studies increasingly include children in the first few years of life and include several TLR-agonists as stimulants, which increases the complexity of analysis; the reason for this being that TLR-dependent vaccine adjuvants have been developed in recent years (29).

The heterogeneous study designs limit conclusions to only small subgroups with comparable study settings. The smallest group was six studies investigating intracellular cytokine expression, of which three led to consistent findings (2, 6, 8–11). First, the frequency of intracellular cytokines in unstimulated samples is generally very low and, therefore, often cannot be analyzed. Second, IFN-γ and TNF-α-producing T cells are detected in varying frequency; however, an age-associated increase was seen in all studies. Finally, expression of other cytokines such as IL-2 and IL-10 has yielded different and conflicting patterns in different studies.

The second-largest group of studies analyzed cytokine profiles in unstimulated plasma or serum samples. Although it could be assumed that this is the most homogenous group as there is no variability from *in vitro* stimulation, the comparison of results from the seven studies in this group is challenging for a number of reasons (3, 13–16, 18). First, many cytokines are expressed at low or undetectable concentrations. For example, a study including children in the first 5 years of life—when immune maturation is expected to be most prominent—did not show an age association of any of the cytokines investigated, while mean concentrations of all cytokines were below 10 pg/ml (3). This fact also has likely influenced analysis of other studies, as separate results and analysis of unstimulated samples are rarely included in the study results. In addition, the studies included show a highly variable age range from infants below 6 months only (16), children below 5 years of age (3), adolescents only (14, 18) or spanning a broad age range (15, 17, 18). Studies including children in a narrow age range commonly did not perform age analysis. Only two studies are of major interest, as they included larger age ranges and analyzed several cytokines (13, 17). Two findings were consistent in both studies, being an age-dependent decrease of sIL-2R and increase of TNF-α. Interestingly, for other cytokines, non-linear patterns, such as uni- or bimodal patterns were described. For example, Sack et al. showed a bimodal pattern for IL-1RA and IL-6 (13). Unfortunately, older studies both using stimulated and unstimulated samples mostly fail to describe non-linear patterns as results were analyzed in larger age groups, which does not allow for the analysis of more complex patterns of cytokine expression (13).

The largest subgroup of studies included in this review comprised 12 studies that analyzed cytokines in stimulated and unstimulated samples (10–12, 19, 20, 22–25, 27, 28). This group of studies is clearly the most heterogeneous, as all laboratory parameters such as type of stimulatory antigen, duration, and condition of incubation and sample storage likely influence cytokine concentrations. The older studies published between 2001 and 2009 are better comparable, as they included larger and similar age ranges and fewer different stimulatory antigens.

Four studies show an age-associated increase of IFN-γ and TNF-α release (10, 11, 19, 28), which is also consistent with the findings from flow-cytometry studies. Interestingly, this age association is not seen in some of the more recent studies, particularly when TLR-agonists instead of mitogens are used as stimulatory antigens and/or when children in the first year of life only were included (25–27). This suggests that age association can only be detected in studies in which the populations have a large-enough age difference. The importance of the age-interval of investigated populations is further supported by a recent publication in Indonesian children showing that the greatest age-associated increase for IFN-γ and TNF-α was seen after the first year of life (28). In addition, all the more recent studies showing no age influence or an age-associated decrease of IFN-γ and TNF-α releases were performed in resource-limited settings. Evidence suggests that cytokine release is influence by continent of origin and in general, cytokine concentrations were lower in African children compared to children from other continents, thereby potentially limiting the detection of any age association in these studies (30). Both IFN-γ and TNF-α are key cytokines produced by cells from the innate and adaptive immune system and involved in the immune response to many pathogens in early childhood (31). The lower expression of these two cytokines in infants and young children has been postulated to be associated with the susceptibility to numerous infections in early childhood and the failure of diagnosis including those cytokines to perform well (4, 31, 32). Importantly, as both cytokines are included in diagnostic assays and being drug targets, developmental changes during childhood need to be accounted for when choosing test cut-offs for dosing of drugs interfering with those cytokines.

Four studies investigated age-associated changes of IL-13 concentrations, of which three showed an age-associated decrease (23, 25, 28). Interestingly, the only study, which was unable to find an IL-13 decrease, was from an affluent country with high incidence of atopic diseases. IL-13 is well known to be a key factor for the development of allergic disease and asthma and, therefore, changes in concentrations of this cytokine are likely influenced by factors other than age and may explain the lacking decrease even in the non-atopic Australian population (33).

Further challenging results are seen for IL-10 concentrations. While all studies using unstimulated samples did not find an age-association 6 of the 11 studies investigating stimulated samples showed an age-associated decrease for some stimulatory antigens (12, 20, 22, 24–26). Interestingly, when different TLR-agonists were used, different studies yielded different levels of stimulation. For example, whereas TLR2/1 and 7/8 stimulations resulted in high concentrations in children in South Africa, Ecuador, and Papua New Guinea; TLR3 and TLR4 stimulation resulted in low concentrations in South African but high concentrations in Papua New Guinean children, which may partially be explained by the choice of the specific TLR antagonist. That notwithstanding, these findings limit interpretability and conclusion on normal development of IL-10 expression. Similar conflicting results in TLR-stimulated samples are seen for IFN-γ, IL-6, and TNF-α.

A further important aspect is that cytokine analysis from cord blood samples seem to have a distinct pattern with comparatively high IL-2, IL-6, and IL-10 concentrations and low IFN-γ, IP-10, and TNF-α concentrations in a number of studies (2, 8, 10, 11, 24, 26). This fact renders studies including cord blood samples only difficult to interpret and, therefore, those were excluded from the analysis in this review.

Further to age, other factors have been described to influence cytokine concentrations such as sex (2, 14, 23), household sanitation standard (23), sport (14), dietary supplements (21) maternal cytokine profile (28), and method of blood sampling (22).

One potential limitation is that in studies describing cytokines patterns in children; age- and/or other associations in healthy children have not been the primary focus of the study and, therefore, the search criteria were unable to detect these studies. To account for this, references of the included papers were meticulously searched to identify additional papers, which were included in the review.

In summary, our current understanding of the development of cytokine expression in childhood is limited. The dynamic development of cytokine expression is clearly shown for some cytokines and needs to be considered when these are included in diagnostic assays or as biomarkers. In addition, for cytokine-targeting agents dose and particular agent may require treatment adjustments for normal when being in the pediatric population. Further research in healthy children is needed particularly for those cytokines considered as disease biomarkers, vaccine efficacy outcome markers, or drug targets.

## Author Contributions

NR conceived and supervised the review, MLD and NR conducted literature research, analyzed data, wrote the main manuscript, and designed figures and tables. MPG wrote and edited the manuscript. All authors discussed the results and implications and commented on the manuscript at all stages.

## Conflict of Interest Statement

None of the authors has any conflict of interest to declare in relation with this manuscript.
